# Effects of chronic heat stress on granulosa cell apoptosis and follicular atresia in mouse ovary

**DOI:** 10.1186/s40104-016-0116-6

**Published:** 2016-09-29

**Authors:** Jieyun Li, Hui Gao, Zhen Tian, Yi Wu, Yingzheng Wang, Yuan Fang, Lu Lin, Ying Han, Shuaishuai Wu, IhteshamUl Haq, Shenming Zeng

**Affiliations:** 1 Laboratory of Animal Embryonic Biotechnology; National Engineering Laboratory for Animal Breeding; Key Laboratory of Animal Genetics, Breeding, and Reproduction of the Ministry of Agriculture, College of Animal Science and Technology, China Agricultural University, Beijing, 100193 China; 2 State Key Laboratory of Reproductive Biology, Institute of Zoology, Chinese Academy of Sciences, Beijing, 100101 China

**Keywords:** Apoptosis, Atresia, Follicle, Granulosa cells, Heat stress, Mice

## Abstract

**Background:**

Heat stress is known to alter follicular dynamics and granulosa cell function and may contribute to the diminished reproductive efficiency commonly observed in mammals during the summer. Although several investigators have studied heat-induced ovarian injury, few reports have focused on the effects of chronic heat stress on ovarian function and the molecular mechanisms through which it induces ovarian injury.

**Methods:**

In Exp. 1, 48 female mice were assigned to a control or heat-stressed treatment. After exposure to a constant temperature of 25 °C for 7, 14, 21 or 28 d (*n* = 6) or to 42 °C for 3 h per d for 7, 14, 21 or 28 d (*n* = 6), the mice were euthanized and their ovaries were analyzed for follicular atresia, granulosa cell apoptosis, changes in the abundance of HSP70 protein and serum concentrations of estradiol. In Exp. 2, the expression of HSP70 and aromatase was quantified in antral follicles cultured in vitro at 37 or 42 °C for 24 h. In Exp. 3, granulosa cells from ovaries maintained at 37 or 41 °C for 2 h were analyzed for their expression of HSP70, Bim, caspase-3 and cleaved caspase-3.

**Results:**

In Exp. 1, body weight and food intake of heat-stressed mice decreased (*P* < 0.05) compared with control mice while the concentration of estradiol in serum was lower (*P* < 0.05) in heat-stressed mice than in control mice. Compared with control mice, the percentage of atretic follicles and the number of antral follicles with severe apoptotic signals were increased (*P* < 0.05) after 21 d of heat-stressed treatment. HSP70 protein was more abundant (*P* < 0.05) in heat-stressed mice than control mice. In Exp. 2, heat stress increased HSP70 and decreased aromatase proteins (*P* < 0.05) in antral follicles. In Exp. 3, TUNEL-positive granulosa cells from heat-stressed ovaries were observed concomitant with a significant increase in HSP70, Bim and cleaved caspase-3 protein.

**Conclusion:**

Heat-stress in mice decrease estradiol in serum and aromatase in antral follicles but increased number of atretic follicles and granulosa cell undergoing apoptosis which may explain the decreased fertility commonly observed in heat-stressed animals.

## Background

Heat stress is known to alter follicular dynamics [1, 2], steroidogenic ability [1, 3], granulosa cell function [3, 4] and oocyte maturation [5, 6] and can contribute to diminished reproductive efficiency commonly observed in mammals during the summer. One of the best characterized responses of mammals to elevated temperatures is that of the so-called heat shock proteins (HSP) [7]. HSPs act as molecular chaperones, assisting in the folding, assembly and disassembly of other proteins [8]. Although a number of HSPs are induced by diverse environmental stressors, one particular family of stress proteins, namely the HSP70s, comprise the major class of proteins induced by elevated temperatures [9].

Estradiol is important in the maintenance of ovarian function [10, 11]. It regulates follicle development and ovarian atresia, inhibits granulosa cell apoptosis and promotes the division and growth of granulosa cells [12, 13]. The estradiol levels in follicles have been shown to be significantly decreased by heat-stress in goats [1] and cattle [14]. Heat stress also decreased estradiol secretion in primary cultures of granulosa cells [15]. However, the effects of chronic heat stress on the concentration of estradiol in blood have rarely been investigated. Granulosa cells are critical for normal ovarian function and synthesize an array of factors required for follicle development [16, 17]. Furthermore, the growth, differentiation, and maturation of oocytes are dependent upon proliferation and differentiation of granulosa cells [18]. Follicular cells in atretic follicles are normally eliminated by apoptosis [19]. Thus, a shift in the balance of the signaling pathway between granulosa cell survival and death might alter the fate of ovarian follicles [20, 21]. Although heat stress increases the susceptibility of rat granulosa cells to apoptosis [3], and heat stress impairs granulosa cell function in mice by diminishing steroid production and inducing apoptosis [15], it has not been determined if chronic heat stress affects granulosa cell apoptosis. Two important protein families that regulate apoptosis are those of the Caspase family and the Bcl-2 family [22]. Caspases participate in two major apoptotic cascades namely the death receptor pathway and the mitochondrial pathway [23, 24]. Moreover, They are the final executor of cell apoptosis and the common downstream end-point of several converging apoptotic pathways [23, 24]. Caspase-3 generally exists in the cytoplasm as a 32-kDa zymogen that is activated at the early stages of apoptosis. The activation of caspase-3 to either one of its catalytically active p17- or p12-subunits has been demonstrated in different cells undergoing apoptosis. Furthermore, several hormones and growth factors regulate granulosa cell apoptosis by inhibiting or activating caspase activity. However, the role of caspase in heat stress-induced granulosa cell apoptosis is unknown.

The Bcl-2 protein family is another major regulator of apoptosis [25]. The balance between pro- and anti-apoptotic Bcl-2 members is believed to be responsible for the regulation of granulosa cell apoptosis and survival [26]. Furthermore, Bim (also known as Bcl-2-related ovarian death gene) promotes apoptosis by binding with anti-apoptotic Bcl-2 family members thereby inducing mitochondrial release of cytochrome c, which subsequently activates caspase-9, caspase-3 and death effector molecules [27]. Bim proteins were also identified in granulosa cells of primordial, primary, secondary, and mature follicles in the mouse [28]. The cellular functions and the relative contribution of Bim to heat stress-induced apoptosis of granulosa cells have not been elucidated.

Although several investigators have studied heat-induced ovarian injury, few reports have focused on the effects of chronic heat stress on ovarian function and the molecular mechanisms through which heat stress induces ovarian injury. Therefore, this study was designed to investigate the effects of chronic heat stress on follicular atresia, granulosa cell apoptosis and the molecular mechanism of heat-induced ovarian injury.

## Methods

Exp. 1 was conducted in the Peking University Laboratory Animal Centre (Peking University, Beijing, China). The protocol for Exp. 1 was reviewed and approved by the Institutional Animal Care and Use Committee at Peking University (Beijing, China). The animal experimentation procedures for Exp. 2 and 3 were approved by the Institutional Animal Care and Use Committee at China Agricultural University (Beijing, China).

### Animals and treatments

Forty-eight, 3-weeks-old female ICR mice were obtained from Vital River Laboratories (Beijing, China). The animals were housed in individual cages in a climate-controlled room at the Peking University Laboratory Animal Center (Beijing, China) under conditions of 12-h light and 12-h dark and 50 % relative humidity. The mice were randomly assigned to either a control or a heat-stressed treatment. After exposure to a constant temperature of 25 °C for 7, 14, 21 or 28 d (Con *n* = 6) or to 42 °C for 3 h per d for 7, 14, 21 or 28 d (HS *n* = 6), the mice were euthanized and their ovaries were analyzed for follicular atresia, granulosa cell apoptosis, changes in the abundance of HSP70 protein and concentrations of estradiol in serum. The body weights and food intake of mice were measured weekly.

### Sampling of blood and serum hormone assays

Mice were euthanized with isoflurane 7, 14, 21 or 28 d after the onset of treatment and blood was collected from the orbital venous plexus. Samples were collected into evacuated serum tubes (BD Vacutainer; BD and Co., Franklin Lakes, NJ) containing clot activator for serum. The blood was centrifuged and the supernatants were stored at −80 °C until assayed for estradiol. The concentrations of estradiol in serum were measured by a mouse-specific radioimmunoassay (RIA) at the Beijing North Institute of Biological Technology (Beijing, China). The sensitivity of the RIA was 4 pg/mL. The inter- and intra-assay coefficients of variation for estradiol were 20 and 15 %, respectively.

### Histological analysis

Following slaughter, 1 ovary from each mouse was fixed in 4 % paraformaldehyde for 16 h at 4 °C followed by processing in paraffin, sectioning, and staining with haematoxylin and eosin. After staining, the follicles were categorized as primordial, primary, secondary, mature, or atretic. Follicles were classified as primordial if they contained an oocyte surrounded by a single layer of flattened follicular cells. They were classified as primary when the flattened cells of the follicles became squamous or cuboidal (the so-called granulosa cells). Follicles were classified as secondary by the presence of a visible follicular antrum. In follicles with a markedly enlarged antrum, the cumulus oophorus was diminished, leaving the free-floating oocyte surrounded by 2 or 3 layers of granulosa cells. After this stage, the follicle bulged outward from the ovary and were classified as mature follicle. Typical interstitial glands and follicles with a shrunken oocyte or with granulosa cells that had begun to disaggregate were categorized as atretic follicles. The total number of antral follicles and atretic follicles per ovary was determined by taking an average of the counts from 3 sections (5 sections apart) cut along the long axis of the entire ovary. The percentage of atretic follicles in the antral follicles was calculated.

### Detection of apoptotic follicles and granulosa cells

Apoptosis was analyzed by TUNEL using the In Situ Cell Death Detection Kit according to the manufacturer instructions (Roche Applied Science, Indianapolis, IN, USA). After deparaffinization and rehydration of tissue sections, they were incubated with 20 μg/mL proteinase K for 15 min at room temperature, quenched with 3 % H_2_O_2_ in PBS for 10 min to block endogenous peroxidase activity, incubated in a humidified chamber with equilibration buffer for 5 min and treated with terminal deoxynucleotidyltransferase for 1 h at 37 °C. Negative control slides were incubated as described above in the absence of terminal deoxynucleotidyltransferase. Thereafter, tissue sections were washed three times with PBS, counter-stained with 0.5 μg/mL Hoechst 33342 (Sigma-Aldrich, St. Louis, MO, USA) in PBS for 5 min, washed 3 times in PBS, and sealed under cover slips with nail varnish. Slides were examined under a Leica fluorescence microscope (Leica DC 200 digital camera; Leica, Wetzlar, Germany). The total number of antral follicles with weak, intermediate or strong apoptotic signals per ovary was determined by taking an average of the counts from 3 sections (5 sections apart) along the longitudinal axis of the entire ovary.

Granulosa cells were washed 3 times with PBS-PVA (polyvinyl alcohol, 0.1 %) and then fixed with 4 % paraformaldehyde at room temperature for 1 h. After fixation, the specimens were incubated in 0.1 % sodium citrate containing 0.5 % Triton X-100 at 4 °C for 1 h, washed three times with PBS-PVA (polyvinyl alcohol, 0.1 %) and incubated in the dark in TUNEL reaction medium for 1 h at 37 °C . Granulosa cells in the positive control mice were treated with 100 U DNase I in 50 mmol/L Tris–HCl, 10 mmol/L MgCl_2_ and 1 mg/mL bovine serum albumin (BSA, pH 7.5) for 1 h before the TUNEL reaction. After the reaction, the cells were counter-stained as previously indicated and mounted with cover slips that were supported by 4 columns of Vaseline and paraffin (9:1). The slides were sealed with nail varnish and examined under a Leica fluorescence microscope (Leica DC 200 digital camera; Leica, Wetzlar, Germany). Ten granulosa cells from each treatment were evaluated for apoptosis and the experiment was repeated 3 times.

### Protein extraction and Western Blot

Western Blot was performed as described previously [29]. In brief, ovaries were homogenized in lysis buffer to obtain protein lysates, and protein concentrations were determined using the Bicinchoninic Acid Assay (Vigorous, Beijing, China). Equal amounts of protein (100 μg) were loaded and separated by electrophoresis on 7.5 % sodium dodecyl sulfate polyacrylamide gels. Separated proteins were transferred to nitrocellulose membranes (BioTraceNT, Pall Corporation, Ann Arbor, MI). The non-specific binding sites were blocked with 5 % non-fat milk in TBS-T (10 mmol/L Tris, 150 mmol/L NaCl and 0.1 % Tween 20, pH 7.5) for 1 h at room temperature. The membranes were then incubated overnight with primary antibodies at 4 °C.

On the following day, the membranes were washed in TBS-T and incubated with secondary antibodies conjugated to horseradish peroxidase (HRP) for 1 h at room temperature. The primary antibodies used included mouse anti-HSP70 (dilution: 1:2,000; Cat. No: N27F3-4; Stress Gen Biotechnologies Corp., Victoria, BC, Canada), rabbit anti-aromatase (dilution: 1:2,000; Cat. No. A7981; Sigma-Aldrich, St. Louis, MO, USA), rabbit anti-Bim (dilution: 1:4,000; Cat. No. C34C5; Cell Signaling Technology, Inc., Danvers, Massachusetts, United State), rabbit anti-caspase-3 (dilution: 1:100; Cat. No. sc-7148; Santa Cruz Biotechnology, Santa Cruz, CA, USA), and rabbit anti-cleaved caspase-3 (dilution: 1:500; Cat. No. 9661; Cell Signaling Technology, Inc., Beverly, MA, USA). The actin antibody (dilution: 1:2,000) was obtained from Abmart Biotechnology (Shanghai, China). The secondary antibodies were goat anti-mouse IgG antibody-HRP (dilution: 1:5,000; Zymed; San Francisco, CA, USA) and goat anti-rabbit IgG-HRP (ZB-5301, dilution: 1:2,000; Zhongshan Biotechnology, Beijing, China). The protein bands were visualized by enhanced Chemiluminescence Detection Reagents (Applygen Technologies Inc., Beijing, China) and captured with X-OMAT BT film (Eastman Kodak Company, Rochester, NY, USA). The films were digitized, and densitometric analysis was performed using ImageJ 1.44p software (National Institutes of Health, Bethesda, MD, USA). The relative intensity of the bands was quantified and normalized to the respective loading control.

### Granulosa cell collection, culture and treatment

Granulosa cells were obtained from the ovaries of CD-1 female mice. In brief, pre-pubertal mice at 19–21 d of age were injected intra-peritoneally with 10 U of equine chorionic gonadotrophin to stimulate follicle growth and the mice were sacrificed at 44 to 46 h after treatment. The ovaries were harvested, washed with sterile PBS and transferred to a culture dish containing 100 μL of αMEM medium supplemented with 10 U/mL recombinant FSH, 3 mg/mL bovine serum albumin, 1 mg/mL bovine fetuin, 5 μg/mL insulin, 5 μg/mL transferrin and 5 ng/mL selenium. Ovarian heat treatment was carried out in either a 37 or 41 °C waterbath for 2 h, then the ovaries were harvested and washed with sterile PBS and transferred to a culture dish containing M199 medium. A hypodermic needle was used to puncture the large antral follicles to release granulosa cells into the medium. The cumulus-oocyte complex and ovarian tissue were discarded under a stereomicroscope. Thereafter, granulosa cells were pelleted by centrifugation (10 min at 500 × *g*), washed three times with PBS and cultured in 6-well plates (5 × 10^6^ cells/well) with 2 mL of DMEM/F12 containing 10 % heat-inactivated fetal bovine serum, 100 U/mL penicillin and 50 mg/mL streptomycin. Cells were incubated in a humidified atmosphere of 5 % CO_2_ at 37 °C for 24 h. Upon completion of the experiment, granulosa cells from each treatment were collected for subsequent analysis by Trypan Blue Stain or snap-frozen for subsequent analysis by TUNEL or Western Blot.

### Granulosa cell viability assay

An aliquot (10 μL) of granulosa cells (about 5 × 10^5^ cells/mL) was combined with 1 μL of 0.4 % Trypan Blue Solution in centrifuge tube, the cells were stained for 3 min and the cell concentration was determined with a Hemocytometer (Shanghai Qiujing Biochemical Reagent and Instrument Company, Shanghai, China). Images were captured immediately using a Leica microscope (Leica DC 200 Digital Camera; Leica, Wetzlar, Germany).

### Follicle collection, culture and treatment

Early antral and pre-ovulatory follicles were collected from CD-1 female mice at 16 d of age as described previously [30]. The dissected ovaries were transferred to a 35-mm Petri dish with 1 or 2 mL of Maintenance Medium (αMEM containing 1 % FBS, 50 U/mL penicillin and 50 μg/mL streptomycin) containing 0.1 % collagenase and 0.1 % DNase, and incubated in an atmosphere of 5 % CO_2_ at 37 °C for 15 min. Thereafter, the follicles were washed and transferred to another 35-mm Petri dish with L15 medium containing 1 % FBS, 50 U/mL penicillin and 50 mg/mL streptomycin. Follicles were isolated by gently flicking the dish or cutting the follicles away from the entire ovary with two hypodermic needles and early antral and pre-ovulatory follicles were then carefully removed at room temperature. One and 3 mL of Maintenance Medium was added to the central well and outer ring of a 60 mm in vitro fertilization (IVF) Petri dish, respectively. Intact follicles were transferred with a pipette to outer ring of the IVF dish, briefly rinsed, and then transferred to the central well. The IVF dish was briefly incubated. Thereafter, follicles were transferred to a 96-well culture plate containing 100 μL of αMEM media supplemented with 10 U/mL recombinant FSH, 3 mg/mL bovine serum albumin, 1 mg/mL bovine fetuin, 5 μg/mL insulin, 5 μg/mL transferrin and 5 ng/mL selenium. The follicles were incubated in a humidified atmosphere of 5 % CO_2_ at 37 °C or 42 °C for 24 h, and then collected and snap-frozen for subsequent analysis by Western Blot to detect the abundance of HSP70 and aromatase.

### Statistical analyses

Data are presented as means ± S.E.M. Statistical significance was analyzed using a one-way ANOVA of variance with *post hoc* Dunnett’s test (SAS Inst. Inc., Cary, NC, USA). Significant differences were set at *P* < 0.05 (*). In Exp. 1, data are expressed as the means from 6 replicates of control and heat-stressed mice.

## Results

### Exp. 1

**Fig. 1:**
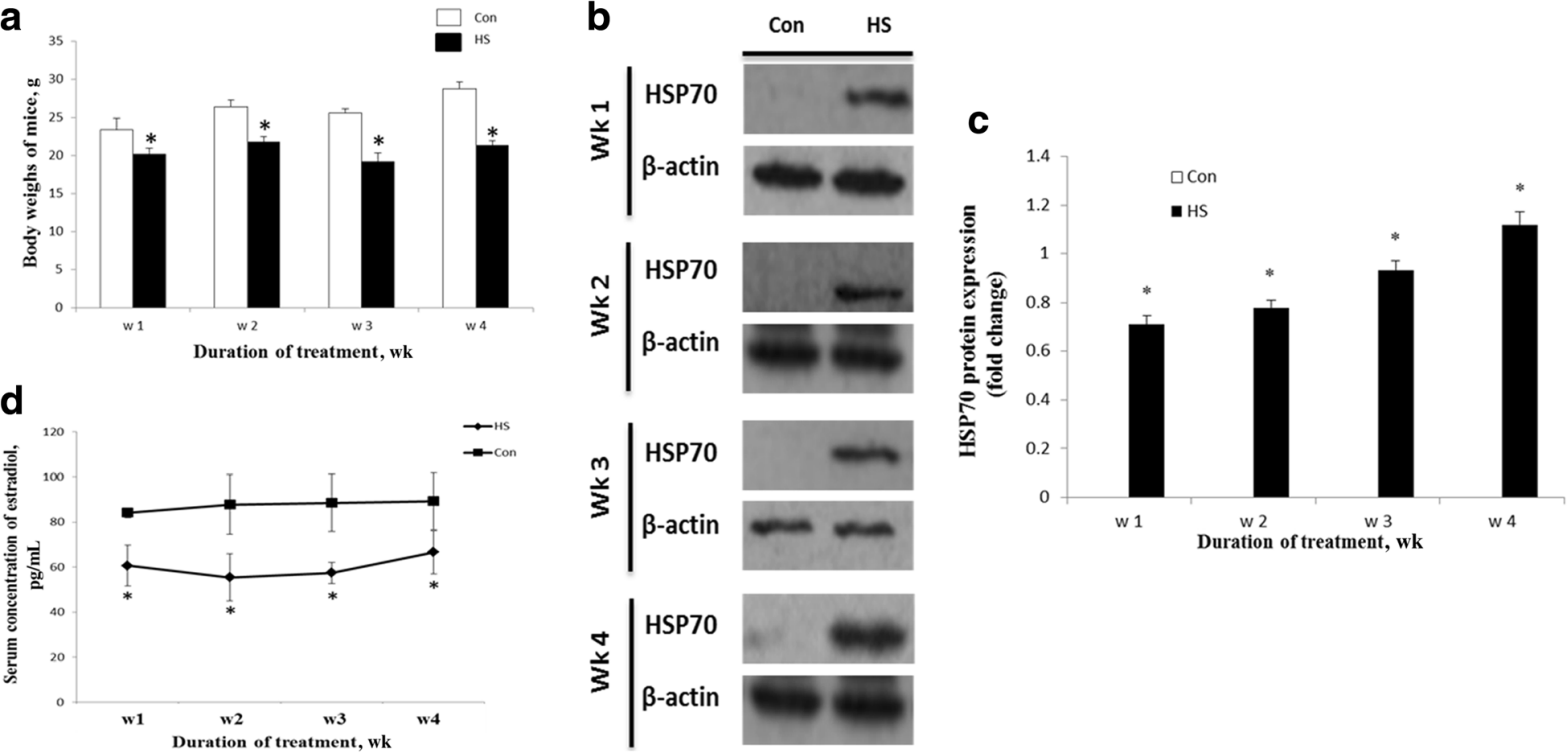
**a** Effects of chronic heat stress on body weights, ovarian HSP70 level and serum estradiol concentration. Effects of heat stress on body weights of control and heat-stressed female mice. **b** and **c** Expression of HSP70 in ovaries of control (Con) and heat-stressed (HS) mice. HSP70 was detected in the lysates of ovaries of mice after each wk of treatment by Western Blot. β-actin was used as a reference. **d** Concentrations of estradiol in serum of heat-stressed mice from d 7 to 28 of treatment. *, indicates *P* <0.05 compared with the control

**Fig. 2:**
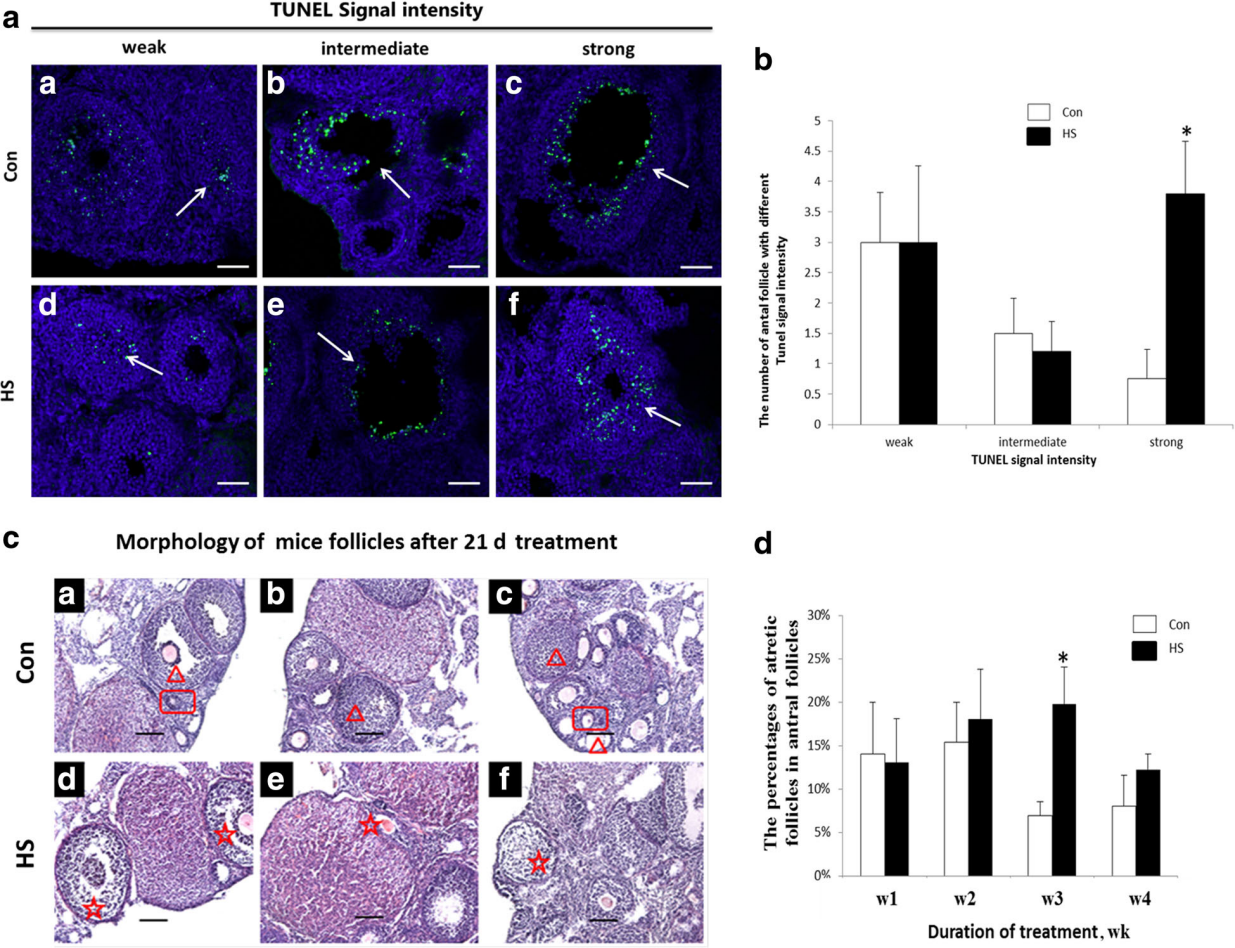
Effects of chronic heat stress on granulosa cells apoptosis and follicular atresia in the ovaries of mice. **a** TUNEL signals intensities in the follicles of ovaries from control (Con) and heat-stressed (HS) mice after 21 d of treatment: (*a*, *d*) follicles with weak apoptotic signals, (*b*, *e*) follicles with intermediate apoptotic signals, (*c*, *f*) follicles with strong apoptotic signals. Bar = 50 μm. **b** Effects of heat stress on the number of antral follicles with different apoptosis signal intensities after 21 d of heat treatment. **c** Morphology of follicles stained with haematoxylin and eosin from ovaries of control and heat-stressed mice. Panels (*a*-*c*) Follicles from ovaries of control mice after 21 d of treatment. Panels (*d*-*f*) follicles from ovaries of heat-stressed mice after 21 d of treatment. The red triangle indicates healthy antral follicles; the red rectangle indicates healthy pre-antral follicles; the red asterisk indicates atretic follicles. Bar = 50 μm. **d** The percentage of atretic follicles in the total population of antral follicles on the ovaries after 4 wk of treatment. *indicates *P* <0.05 compared with the control

The effects of chronic heat stress on the serum estradiol, granulosa cell apoptosis and folliclular atresia in mouse ovaries are showed in Figs. 1 and 2. As shown in Fig. 1a, the body weights of heat-stressed mice decreased (*P* < 0.05) after 7, 14, 21 or 28 d of treatment compared with control mice. With increasing time on experiment, the body weights of control mice increased but the body weights of heat-stressed mice did not. Food intake was also reduced in mice after 21 d of heat stress (data not shown).

The Western Blot results showed that HSP70 was not expressed in the ovary of control mice. However, HSP70 was highly expressed in the ovary of heat-stressed mice 7, 14, 21 or 28 d after treatment (Fig. 1b and c). The concentration of estradiol in serum decreased (*P* < 0.05) after 7, 14, 21 and 28 d of heat stress (Fig. 1d). The effects of heat stress on apoptosis of granulosa cells in antral follicles after 21 d of treatment are illustrated in Fig. 2a and b. TUNEL showed that there was no difference in the number of antral follicles with mild or moderate apoptotic signals between the control and heat stress mice. However, the number of antral follicles with severe apoptotic signals increased (*P* < 0.05) in the heat stress mice compared with the control mice (Fig. 2b).

The percentage of atretic follicles relative to all antral follicles is illustrated in Fig. 2c and d. The percentage of atretic follicles in the antral follicles of the heat stress mice was greater (*P* < 0.05) than for control mice 21 d after initiation of treatments (Fig. 2c).

### Exp. 2

**Fig. 3:**
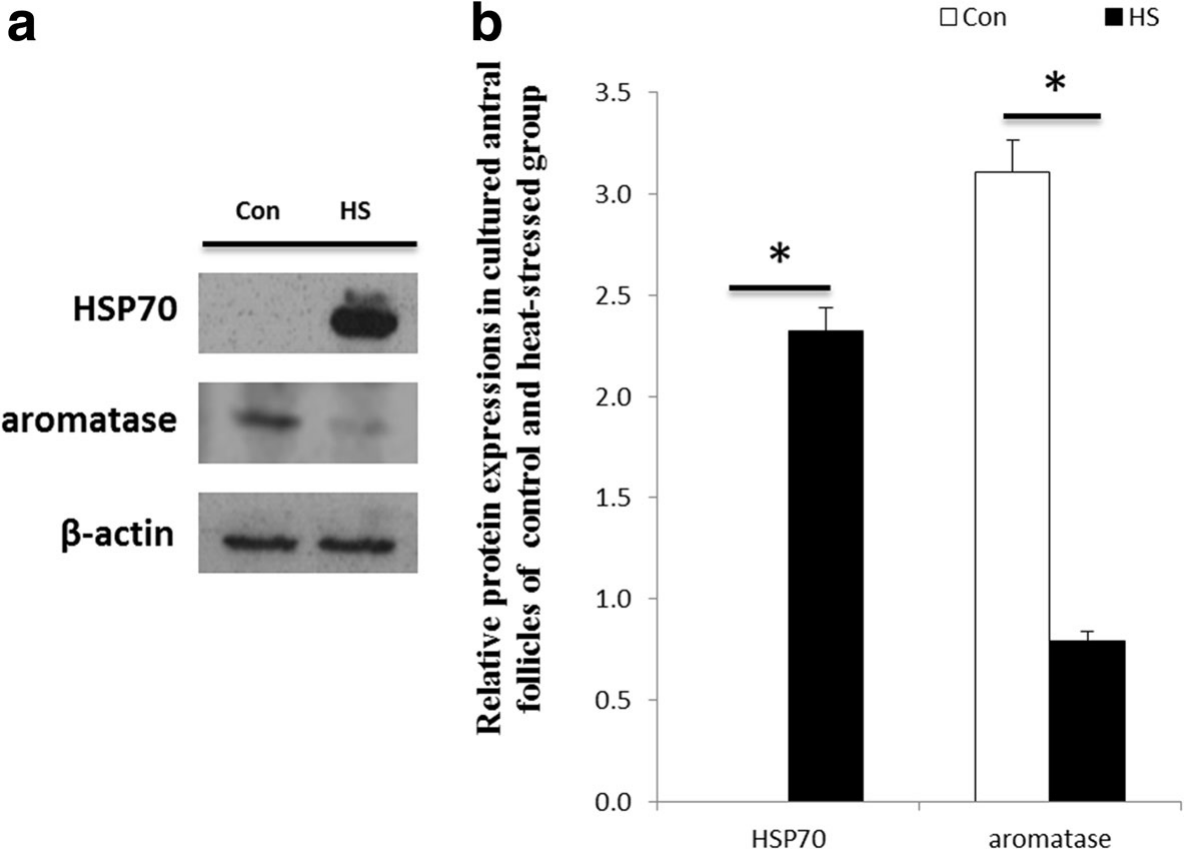
Effects of heat stress on expression of HSP70 and aromatase in cultured antral follicles obtained from mice. **a** Effect of heat stress on the expression of HSP70 and aromatase in cultured antral follicles. Antral follicles were cultured at 37 (Con) or 42 °C (HS) for 24 h. HSP70 and aromatase proteins were analyzed by Western Blotting. **b** Relative abundance of HSP70 and aromatase proteins in antral follicles cultured at 37 (Con) or 42 °C (HS) for 24 h. Proteins were normalized with β-actin. *indicates *P* < 0.05 compared with the control

The effects of heat stress on the abundance of HSP70 and aromatase for in vitro cultured antral follicles are shown in Fig. 3a and b. HSP70 was not expressed in the follicles of control mice. However, HSP70 was highly expressed in the follicles of heat-stressed mice. The expression of aromatase in heat-stressed mice was also decreased (*P* < 0.05) compared with the control mice.

### Exp. 3

**Fig. 4:**
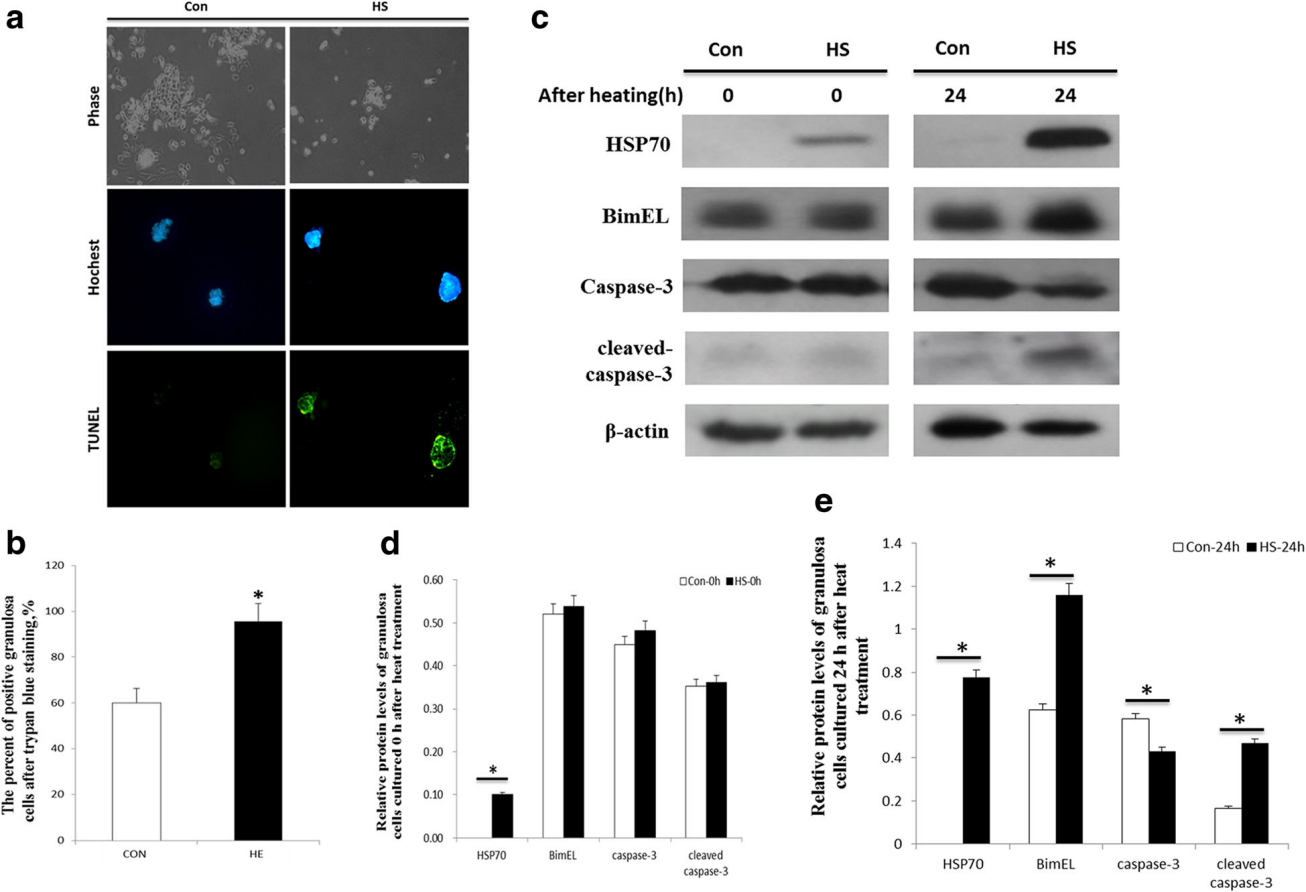
**a** Effects of heat stress on apoptosis in ovarian granulosa cells. Representative photomicrographs of Hoechst and TUNEL-stained granulosa cells from ovaries of control and heat-stressed mice. Ovarian heat treatment was carried out in a 37 (Con) or 41 °C (HS) water bath for 2 h. After heat stress, granulosa cells were collected from the ovaries and cultured for another 24 h. **b** The percentage of trypan blue-positive granulosa cells from the ovaries of mice in the control (Con) and heat stress (HS) treatments. **c** The ovaries were exposed to a 37 (Con) or 41 °C (HS) water bath for 2 h and then the granulosa cells were collected and cultured for either 0 or 24 h. Thereafter, the cells were collected and the expression of HSP70, Bim, caspase-3 and cleaved caspase-3 were detected in the cell lysates. **d** Relative protein levels of granulosa cells before heat treatment. **e** Relative protein levels of granulosa cells 24 h after heat treatment. *indicates *P* < 0.05 compared with the control

The effects of heat stress on apoptosis of granulosa cells in ovaries are shown in Fig. 4. The ovaries were exposed to temperatures of 37 or 41 °C in a water bath for 2 h and then granulosa cells were harvested and cultured for another 24 h. Compared with the control mice, granulosa cells of the heat-stressed mice had a slower rate of growth after heat stress and an increase in apoptosis compared with granulosa cells from control mice (Fig. 4a). Trypan Blue staining was used to verify granulosa cell viability. The percentage of positive granulosa cells from heat-stressed ovaries was greater (*P* < 0.05) than for granulosa cells from control ovaries (Fig. 4b).

The effects of heat stress on the abundance of different proteins in granulosa cells of heat- stressed mice are shown in Fig. 4c. After ovaries were incubated for 2 h at 37 °C or 41 °C, there were no differences in Bim, caspase-3 or cleaved caspase 3 proteins in granulosa cells (Fig. 4d). The HSP70 level increased in granulosa cells (Fig. 4d). However, 24 h after treatment, the abundance of HSP70 (*P* < 0.05), Bim (*P* < 0.05) and cleaved caspase 3 (*P* < 0.05) proteins increased in granulosa cells from ovaries subjected to 41 °C compared to 37 °C while the abundance of caspase-3 decreased (*P* < 0.05; Fig. 4e).

## Discussion

Our results illustrated that chronic heat stress reduced concentrations of estradiol in serum and increased apoptosis of granulosa cells and follicle atresia. Our results also indicated that caspase-3 and Bim are involved in the heat stress-induced apoptosis of granulosa cells.

Causes of reduced fertility during the summer include changes in follicular dynamics and steroidogenic activity [1, 3], lower oocyte competence [5, 6], a reduction in the expression of estrus [31] and early embryonic death [32, 33]. To our knowledge, there are no studies reporting changes in the serum estradiol, granulosa cell apoptosis and follicular atresia after chronic heat stress.

In this study, we observed a significant reduction in body weight and food intake in mice (data not shown) after 21 d of heat stress, which was indicative of reduced energy intake and a nutritional imbalance. Previous studies have indicated that nutrition has an important influence on reproductive function [34, 35]. Acute nutrient restriction reduced the size of the dominant follicle, and resulted in the absence of a pre-ovulatory increase in estradiol and the ovulatory surge of LH, resulting in ovulatory failure in beef heifers [36]. On the other hand, a short period of improved nutrition can stimulate ovulation rate in sheep [37]. For example, lupin grain supplements fed to ewes for 4 to 6 d before luteolysis increased the ovulation rate [37]. In the present study, we observed an increase in follicular atresia after 21 d of heat stress. Therefore, the indirect effects of low energy and a nutritional imbalance cannot be ignored compared with the direct effects of heat stress on follicular function.

Our results showed that the inducible HSP70 were increased in the ovaries of mice after 7, 14, 21 and 28 d of heat stress, in cultured follicles after 24 h of heat stress, and in cultured granulosa cells from ovaries after 2 h of heat stress. These findings confirm the results of studies using porcine ovaries [38, 39] and Chinese hamster ovary cells [40]. It is suggested that the induction of HSP70 in ovary cells under heat stress is a protective mechanism. HSP70, the major heat stress-inducible protein, protects cells from heat stress-induced apoptosis and affects the apoptotic pathway at the levels of both Cytochrome C release and initiator caspase activation [41]. In the present study, there were increases in the apoptosis of granulosa cells from ovarian follicles of 21-d heat-stressed mice and heat stressed ovaries. We suggest that the induction of HSP70 occurs to reduce apoptosis of granulosa cells induced by heat stress.

It has been reported that acute heat stress decreased the concentrations of estradiol in serum and follicular fluid in cattle [42–45] and goats [1]. In the present study, serum concentrations of estradiol were significantly decreased after 7, 14, 21 and 28 d of heat stress. The decrease in serum estradiol could be related to the significant decrease in estradiol concentration in the fluid of follicles and reflect a decrease in the steroidogenic capacities of the follicular cells caused by heat stress. Estrogen production is stimulated by the binding of FSH to its receptors on the granulosa cell membrane which activates the aromatase enzyme that converts testosterone to estradiol [46, 47]. It is reported that heat stress inhibits the expression of gonadotropin receptors in granulosa cells [3]. In our study, the abundance of aromatase in cultured follicles decreased after heat stress. The decreases in aromatase activity and estradiol concentrations are in agreement with a previous report that the estradiol concentration and aromatase activity in the dominant follicle in cattle are significantly lower in the summer than in the autumn [14]. Therefore, we suggest that heat stress decreases gonadotropin receptor expression and aromatase activity, suppresses estradiol synthesis in estrogenic follicles and induces a decrease in the production of estradiol.

In the present study, heat stress for 21 d significantly increased the number of apoptotic granulosa cells and there was an increase in the apoptosis of cultured granulosa cells from follicles incubated at 41 °C for 2 h compared with control follicles. These results are in agreement with those in previous studies showing that heat stress induced apoptosis in granulosa cells incubated at 40 or 43 °C in mice and sheep [4, 21], as well as in granulosa cells from heat-stressed follicles harvested 48 h after PMSG injection in rats [3]. Therefore, it is suggested that heat stress induced ovarian granulosa cells apoptosis. Our data also indicates that atresia of antral follicles increases after 21 d of heat stress in mice. These results are in agreement with those in previous studies showing that heat stress during follicular recruitment suppresses subsequent follicle growth to ovulation [1] and reduces the size of the dominant follicle by d 8 in cattle [14] and between d 11 and 21 of the estrous cycle in cows and heifers [44, 45]. It is suggested that heat stress compromises follicular development and promotes antral follicle atresia.

How exactly heat stress induces granulosa cell apoptosis and impacts follicular development is not well understood. One mechanism may be a decrease in the production of estradiol. Granulosa cell apoptosis results from the lack of survival factors such as estradiol [12, 13]. Estrogens inhibit ovarian granulosa cell apoptosis in early antral and pre-antral follicles [12] and estradiol inhibits the activation of endogenous endonucleases and promotes the division and growth of granulosa cells [48]. Therefore, the increase in granulosa cell apoptosis after heat stress may be induced by a decrease in estradiol production. On the other hand, estrogen is essential for folliculogenesis beyond the antral stage of development, as studies with estrogen receptor knockout and estrogen-depleted mice have shown [49]. A rapid decline of estradiol in plasma is an early sign of atresia in antral follicles [50] but most follicles do not reach the ovulatory stage and instead become atretic owing to apoptosis of the granulosa cell lining [25, 51]. Therefore, it is suggested that heat stress decreases estradiol production, enhances susceptibility to apoptosis of granulosa cells and causes antral follicle atresia.

Our findings indicate that the expression of caspase-3, cleaved caspase-3 and Bim in granulosa cells was similar in the two treatments at the start of the experiment. HSP70 was not expressed in granulosa cells from control ovaries but increased in granulosa cells from heat-stressed ovaries. Our results indicate that it takes more than 2 h for caspase-3 and Bim to respond to heat stress. After culturing granulosa cells for 24 h, the abundance of HSP70, cleaved caspase-3 and Bim increased, but caspase-3 decreased in the heat-stressed treatment. In addition, there was a significant increase in apoptosis of granulosa cells in the heat-stressed treatment compared with control mice. We suggest that the pro-apoptotic protein Bim and apoptosis executor caspase-3 are connected with heat-induced apoptosis of granulosa cells, and the decrease in the caspase-3 level and the increase in the cleaved caspase-3 level may be due to the conversion of caspase-3 to cleaved caspase-3.

The molecular mechanism of heat shock-induced apoptosis of granulosa cells is not clear. Previous studies have indicated that heat-induced apoptosis of mouse granulosa cells via the mitochondrial pathway involves caspase-3 [21], and that the mRNA levels of Bim and caspase-3 increase in atretic follicles from porcine ovaries [52]. It has also been proposed that Bim is essential to heat shock-induced cell death which induces mitochondrial outer membrane permeabilization and the loss of mitochondrial inner membrane potential in a BAX/BAK-dependent manner that is antagonized by anti-apoptotic BCL-2 family members in MEFs and human Jurkat T cells [53]. In addition, cells utilize various BH3-only family members to integrate a variety of cellular stressors, all of which induce mitochondrial outer membrane permeabilization, apoptosome assembly, caspase activation, and cell death [54]. Furthermore, the increase in Bim observed in the present study may mediate heat shock-induced apoptosis of granulosa cells, and induce mitochondrial outer membrane permeabilization, apoptosome assembly and caspase activation. Whether Bim mediates heat shock-induced apoptosis through a BAX/BAK-dependent pathway that is antagonized by antiapoptotic BCL-2 family members in heat stress-induced apoptosis of granulosa cells should be explored in the future.

## Conclusion

In conclusion, chronic heat stress reduced the concentrations of estradiol in serum and increased apoptosis of granulosa cells and follicle atresia. Caspase-3 and Bim were involved in the heat stress-induced apoptosis of granulosa cells. Moreover, it is likely that reduced energy intake and a nutritional imbalance as a consequence of reduced food intake contributed to chronic heat stress-induced ovarian dysfunction. Further studies are needed to explain the precise mechanisms for these findings.
